# 2-(2*H*-Benzotriazol-2-yl)-6-[(dicyclo­hexyl­amino)­meth­yl]-4-(2,4,4-trimethyl­pentan-2-yl)phenol

**DOI:** 10.1107/S1600536812038962

**Published:** 2012-09-19

**Authors:** Ming-Jen Chen, Ban-Hsin Wu, Chen-Yu Li, Chia-Her Lin, Bao-Tsan Ko

**Affiliations:** a Department of Applied Cosmetology and Graduate Institute of Cosmetic Science, Hungkuang University, Taichung City 443, Taiwan; b Department of Chemistry, Chung Yuan Christian University, Chung-Li 320, Taiwan

## Abstract

In the title mol­ecule, C_33_H_48_N_4_O, the dihedral angle between the mean planes of the benzotriazole ring system [maximun deviation = 0.038 (2) Å] and the phenol ring is 16.6 (2)°. There is an intra­molecular O—H⋯N hydrogen bond between the phenol and benzotriazole groups.

## Related literature

For background information and potential applications of the title compound, see: Chmura *et al.* (2006 ▶); Gendler *et al.* (2006 ▶); Li *et al.* (2011 ▶). For a related structure: see: Li *et al.* (2009 ▶).
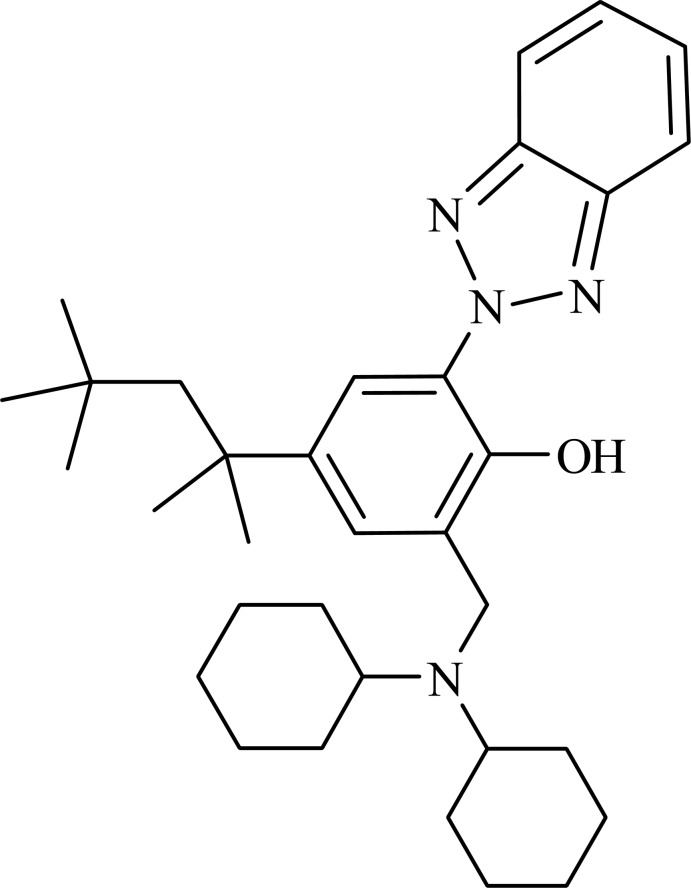


## Experimental

### 

#### Crystal data

C_33_H_48_N_4_O
*M* *_r_* = 516.75
Monoclinic, 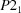

*a* = 11.9975 (2) Å
*b* = 9.9620 (2) Å
*c* = 12.7511 (2) Å
β = 94.468 (1)°
*V* = 1519.37 (5) Å^3^
*Z* = 2
Mo *K*α radiation
μ = 0.07 mm^−1^
*T* = 296 K
0.52 × 0.45 × 0.23 mm

#### Data collection

Bruker APEXII CCD diffractometer
Absorption correction: multi-scan (*SADABS*; Bruker, 2008 ▶) *T*_min_ = 0.968, *T*_max_ = 0.981
13765 measured reflections
6650 independent reflections
5895 reflections with *I* > 2σ(*I*)
*R*_int_ = 0.015

#### Refinement

*R*[*F* ^2^ > 2σ(*F* ^2^)] = 0.036
*wR*(*F* ^2^) = 0.101
*S* = 1.01
6650 reflections
347 parameters
1 restraint
H-atom parameters constrained
Δρ_max_ = 0.15 e Å^−3^
Δρ_min_ = −0.15 e Å^−3^

### 

Data collection: *APEX2* (Bruker, 2008 ▶); cell refinement: *SAINT* (Bruker, 2008 ▶); data reduction: *SAINT*; program(s) used to solve structure: *SHELXS97* (Sheldrick, 2008 ▶); program(s) used to refine structure: *SHELXL97* (Sheldrick, 2008 ▶); molecular graphics: *SHELXTL* (Sheldrick, 2008 ▶); software used to prepare material for publication: *SHELXTL*.

## Supplementary Material

Crystal structure: contains datablock(s) I, global. DOI: 10.1107/S1600536812038962/lh5530sup1.cif

Structure factors: contains datablock(s) I. DOI: 10.1107/S1600536812038962/lh5530Isup2.hkl

Supplementary material file. DOI: 10.1107/S1600536812038962/lh5530Isup3.cml

Additional supplementary materials:  crystallographic information; 3D view; checkCIF report

## Figures and Tables

**Table 1:** Hydrogen-bond geometry (Å, °)

*D*—H⋯*A*	*D*—H	H⋯*A*	*D*⋯*A*	*D*—H⋯*A*
O—H0*A*⋯N1	0.85	1.88	2.618 (2)	145

**Fig. 1.**
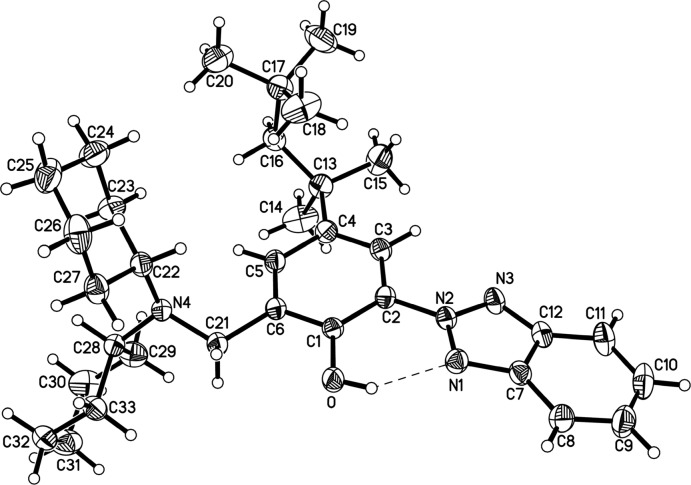
The molecular structure of (I) with displacement ellipsoids drawn at the 30% probability level.

**Fig. 2.**
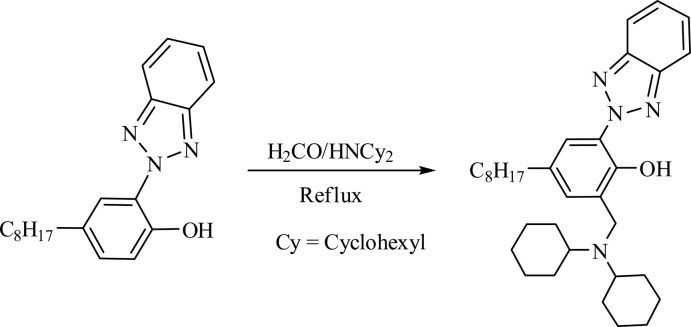
The synthetic procedure.

## supplementary crystallographic information

### Comment

Over the past decade, there has been considerable attention drawn to the development of amine-phenolate titanium alkoxides, due mainly to their applications in polymer preparations of well defined polyesters such as poly(ε-caprolactone) (PCL) and poly(lactide) (PLA) (Chmura *et al.*, 2006 & Gendler *et al.*, 2006). Such amine-phenolate type ligands can be easily synthesized *via* a Mannich condensation from a secondary (or primary) amine, paraformaldehyde, and 2, 4-di-substitute-phenol under reflux conditions. For instance, our group has successfully synthesized and structural characterized amine-phenolate ligands derived from benzotriazole phenoxide (BTP) ligands, and zinc complexes bearing such ligands have been demonstrated to catalyze the ε-caprolactone and β-butyrolactone polymerizations with good catalytic activities in a controlled fashion (Li *et al.*, 2011). However, no amino-BTP ligand with sterically bulky pendant arm has been isolated to date. Herein, we present the synthesis and crystal structure of the title compound, (I), a potential ligand for the preparations of group 4 complexes.

The molecular structure of (I) is composed of a benzotriazole-phenol moiety and the dicyclohexylamino functionalized group (Fig. 1). The dihedral angle between the mean planes of the benzotriazole unit (maximun deviation 0.038 (2)Å for C12) and the benzene ring of the phenoxy group is 16.6 (2)°. There is an intramolecular O—H···N hydrogen bond between the phenol and benzotriazole groups (Table 1). The distance of N1···H is substantially shorter than the van der Waals distance of 2.75 Å for the N and H distance. It is interesting to note that the six-membered ring (O/C1/C2/N2/N1/H0A) formed from the O—H···N hydrogen-bond is almost planar with a mean deviation of 0.041 (2) Å. The bond distances of benzotriazol-phenolate group are similar to those found in the crystal structure of 2-(2*H*-benzotriazol-2-yl)-6-((diethylamino)methyl)-4-methylphenol (Li *et al.*, 2009).

### Experimental

The title compound was synthesized by the following procedure (Fig. 2): To a mixture of paraformaldehyde (1.20 g, 40.0 mmol) and dicyclohexylamine (8 ml, 40.0 mmol) was added 2-(2*H*-benzotriazol-2-yl)-4-(2,4,4-trimethylpentan-2-yl)phenol (3.23 g, 10.0 mmol). The resulting mixture was heated at 383K for 1 day and then dried under reduced pressure to yield the oily residue. The residue was separated by chromatography over silica gel and eluted with hexane/ethyl acetate (95/5) to afford 4.38 g of the title compound in 85% yield. Colorless crystals were obtained from the saturated hexane solution.

### Refinement

The H atoms were placed in idealized positions and constrained to ride on their parent atoms, with C—H = 0.93 Å with *U*_iso_(H) = 1.2 *U*_eq_(C) for benzene hydrogens; 0.96 Å with *U*_iso_(H) = 1.5 *U*_eq_(C) for CH_3_ group; 0.97 Å with *U*_iso_(H) = 1.2 *U*_eq_(C) for CH_2_ group; 0.98 Å with *U*_iso_(H) = 1.2 *U*_eq_(C) for CH group; O—H = 0.85 Å with *U*_iso_(H) = 1.5 *U*_eq_(O).

### Crystal data

**Table:**

C_33_H_48_N_4_O	*F*(000) = 564
*M**_r_* = 516.75	*D*_x_ = 1.130 Mg m^−^^3^
Monoclinic, *P*2_1_	Mo *K*α radiation, λ = 0.71073 Å
Hall symbol: P 2yb	Cell parameters from 9957 reflections
*a* = 11.9975 (2) Å	θ = 2.4–28.2°
*b* = 9.9620 (2) Å	µ = 0.07 mm^−^^1^
*c* = 12.7511 (2) Å	*T* = 296 K
β = 94.468 (1)°	Columnar, yellow
*V* = 1519.37 (5) Å^3^	0.52 × 0.45 × 0.23 mm
*Z* = 2	

### Data collection

**Table:**

Bruker APEXII CCD diffractometer	6650 independent reflections
Radiation source: fine-focus sealed tube	5895 reflections with *I* > 2σ(*I*)
Graphite monochromator	*R*_int_ = 0.015
Detector resolution: 8.3333 pixels mm^-1^	θ_max_ = 28.3°, θ_min_ = 1.7°
φ and ω scans	*h* = −15→13
Absorption correction: multi-scan (*SADABS*; Bruker, 2008)	*k* = −13→13
*T*_min_ = 0.968, *T*_max_ = 0.981	*l* = −17→11
13765 measured reflections	

### Refinement

**Table:**

Refinement on *F*^2^	Primary atom site location: structure-invariant direct methods
Least-squares matrix: full	Secondary atom site location: difference Fourier map
*R*[*F*^2^ > 2σ(*F*^2^)] = 0.036	Hydrogen site location: inferred from neighbouring sites
*wR*(*F*^2^) = 0.101	H-atom parameters constrained
*S* = 1.01	*w* = 1/[σ^2^(*F*_o_^2^) + (0.060*P*)^2^ + 0.080*P*] where *P* = (*F*_o_^2^ + 2*F*_c_^2^)/3
6650 reflections	(Δ/σ)_max_ < 0.001
347 parameters	Δρ_max_ = 0.15 e Å^−^^3^
1 restraint	Δρ_min_ = −0.15 e Å^−^^3^

### Special details

**Table:**

Geometry. All e.s.d.'s (except the e.s.d. in the dihedral angle between two l.s. planes) are estimated using the full covariance matrix. The cell e.s.d.'s are taken into account individually in the estimation of e.s.d.'s in distances, angles and torsion angles; correlations between e.s.d.'s in cell parameters are only used when they are defined by crystal symmetry. An approximate (isotropic) treatment of cell e.s.d.'s is used for estimating e.s.d.'s involving l.s. planes.
Refinement. Refinement of *F*^2^ against ALL reflections. The weighted *R*-factor *wR* and goodness of fit *S* are based on *F*^2^, conventional *R*-factors *R* are based on *F*, with *F* set to zero for negative *F*^2^. The threshold expression of *F*^2^ > σ(*F*^2^) is used only for calculating *R*-factors(gt) *etc*. and is not relevant to the choice of reflections for refinement. *R*-factors based on *F*^2^ are statistically about twice as large as those based on *F*, and *R*- factors based on ALL data will be even larger.

### Fractional atomic coordinates and isotropic or equivalent isotropic displacement parameters (Å^2^)

**Table:**

	*x*	*y*	*z*	*U*_iso_*/*U*_eq_	
O	0.53426 (8)	0.35702 (10)	0.14005 (8)	0.0496 (3)	
H0A	0.5588	0.2840	0.1162	0.074*	
N1	0.68618 (9)	0.17687 (12)	0.10591 (9)	0.0443 (3)	
N2	0.76242 (9)	0.26671 (11)	0.14046 (8)	0.0395 (2)	
N3	0.86727 (9)	0.24269 (13)	0.11847 (10)	0.0484 (3)	
N4	0.45573 (9)	0.71136 (12)	0.29380 (9)	0.0432 (3)	
C1	0.62072 (10)	0.42880 (13)	0.18658 (10)	0.0380 (3)	
C2	0.73240 (11)	0.38923 (13)	0.19034 (10)	0.0380 (3)	
C3	0.81709 (11)	0.46880 (15)	0.23714 (11)	0.0424 (3)	
H3B	0.8907	0.4389	0.2393	0.051*	
C4	0.79395 (11)	0.59171 (14)	0.28062 (11)	0.0430 (3)	
C5	0.68100 (11)	0.63126 (15)	0.27571 (11)	0.0438 (3)	
H5A	0.6633	0.7138	0.3041	0.053*	
C6	0.59560 (11)	0.55311 (14)	0.23071 (10)	0.0410 (3)	
C7	0.74579 (12)	0.08412 (14)	0.05639 (11)	0.0432 (3)	
C8	0.71115 (15)	−0.03181 (16)	0.00004 (13)	0.0550 (4)	
H8A	0.6372	−0.0605	−0.0043	0.066*	
C9	0.79132 (17)	−0.10002 (18)	−0.04794 (15)	0.0666 (5)	
H9A	0.7710	−0.1761	−0.0871	0.080*	
C10	0.90369 (18)	−0.05918 (18)	−0.04017 (16)	0.0738 (5)	
H10A	0.9555	−0.1097	−0.0738	0.089*	
C11	0.93929 (16)	0.05155 (18)	0.01481 (16)	0.0672 (5)	
H11A	1.0140	0.0775	0.0196	0.081*	
C12	0.85784 (12)	0.12549 (15)	0.06446 (12)	0.0463 (3)	
C13	0.88502 (12)	0.68861 (16)	0.32557 (12)	0.0504 (3)	
C14	0.8818 (2)	0.8105 (2)	0.25089 (16)	0.0819 (6)	
H14A	0.8926	0.7808	0.1808	0.123*	
H14B	0.9402	0.8722	0.2739	0.123*	
H14C	0.8107	0.8545	0.2515	0.123*	
C15	1.00213 (14)	0.6252 (2)	0.32593 (18)	0.0790 (6)	
H15A	1.0171	0.6020	0.2553	0.118*	
H15B	1.0051	0.5458	0.3687	0.118*	
H15C	1.0572	0.6883	0.3540	0.118*	
C16	0.86199 (12)	0.74430 (14)	0.43540 (12)	0.0464 (3)	
H16A	0.9112	0.8209	0.4478	0.056*	
H16B	0.7864	0.7796	0.4286	0.056*	
C17	0.87178 (14)	0.66127 (16)	0.53714 (13)	0.0556 (4)	
C18	0.8127 (2)	0.5252 (2)	0.52482 (16)	0.0873 (7)	
H18A	0.7361	0.5387	0.4994	0.131*	
H18B	0.8157	0.4806	0.5917	0.131*	
H18C	0.8494	0.4710	0.4756	0.131*	
C19	0.99370 (19)	0.6403 (3)	0.5801 (2)	0.0942 (7)	
H19A	0.9956	0.5880	0.6435	0.141*	
H19B	1.0282	0.7258	0.5950	0.141*	
H19C	1.0336	0.5938	0.5287	0.141*	
C20	0.81634 (19)	0.7435 (2)	0.62047 (15)	0.0792 (6)	
H20A	0.7380	0.7530	0.6001	0.119*	
H20B	0.8504	0.8306	0.6265	0.119*	
H20C	0.8259	0.6982	0.6870	0.119*	
C21	0.47483 (12)	0.60067 (16)	0.22229 (13)	0.0498 (4)	
H21A	0.4545	0.6293	0.1506	0.060*	
H21B	0.4267	0.5262	0.2377	0.060*	
C22	0.43782 (12)	0.66512 (14)	0.40017 (11)	0.0444 (3)	
H22A	0.4835	0.5841	0.4118	0.053*	
C23	0.48267 (14)	0.76647 (17)	0.48255 (12)	0.0549 (4)	
H23A	0.4405	0.8493	0.4736	0.066*	
H23B	0.5602	0.7862	0.4720	0.066*	
C24	0.47455 (17)	0.7143 (2)	0.59412 (13)	0.0689 (5)	
H24A	0.5231	0.6370	0.6057	0.083*	
H24B	0.4999	0.7833	0.6441	0.083*	
C25	0.35640 (17)	0.6751 (2)	0.61270 (15)	0.0741 (5)	
H25A	0.3094	0.7544	0.6091	0.089*	
H25B	0.3548	0.6370	0.6826	0.089*	
C26	0.31068 (16)	0.57374 (19)	0.53183 (16)	0.0701 (5)	
H26A	0.3524	0.4906	0.5410	0.084*	
H26B	0.2332	0.5548	0.5429	0.084*	
C27	0.31846 (12)	0.62495 (17)	0.42101 (13)	0.0544 (4)	
H27A	0.2697	0.7021	0.4095	0.065*	
H27B	0.2927	0.5556	0.3715	0.065*	
C28	0.38601 (11)	0.82172 (14)	0.24991 (11)	0.0434 (3)	
H28A	0.3660	0.8762	0.3096	0.052*	
C29	0.45310 (15)	0.91286 (19)	0.18167 (14)	0.0638 (4)	
H29A	0.5201	0.9429	0.2225	0.077*	
H29B	0.4761	0.8619	0.1222	0.077*	
C30	0.3865 (2)	1.0342 (2)	0.14153 (18)	0.0806 (6)	
H30A	0.3721	1.0917	0.2004	0.097*	
H30B	0.4301	1.0851	0.0944	0.097*	
C31	0.27623 (18)	0.9943 (2)	0.08383 (16)	0.0752 (5)	
H31A	0.2334	1.0744	0.0648	0.090*	
H31B	0.2906	0.9477	0.0195	0.090*	
C32	0.20860 (15)	0.90448 (19)	0.15035 (14)	0.0635 (4)	
H32A	0.1413	0.8759	0.1093	0.076*	
H32C	0.1864	0.9546	0.2105	0.076*	
C33	0.27546 (13)	0.78174 (17)	0.18845 (13)	0.0530 (4)	
H33B	0.2915	0.7271	0.1285	0.064*	
H33A	0.2312	0.7282	0.2334	0.064*	

### Atomic displacement parameters (Å^2^)

**Table:**

	*U*^11^	*U*^22^	*U*^33^	*U*^12^	*U*^13^	*U*^23^
O	0.0346 (5)	0.0511 (6)	0.0625 (6)	0.0022 (4)	−0.0005 (4)	−0.0172 (5)
N1	0.0402 (6)	0.0436 (6)	0.0494 (6)	−0.0006 (5)	0.0055 (5)	−0.0046 (5)
N2	0.0327 (5)	0.0423 (6)	0.0438 (6)	0.0033 (5)	0.0057 (4)	−0.0005 (5)
N3	0.0350 (6)	0.0490 (7)	0.0619 (7)	0.0069 (5)	0.0089 (5)	−0.0011 (6)
N4	0.0367 (6)	0.0482 (6)	0.0443 (6)	0.0061 (5)	0.0012 (5)	−0.0095 (5)
C1	0.0322 (6)	0.0442 (7)	0.0377 (6)	0.0002 (5)	0.0037 (5)	−0.0015 (6)
C2	0.0363 (7)	0.0394 (7)	0.0391 (6)	0.0018 (5)	0.0077 (5)	−0.0010 (5)
C3	0.0310 (7)	0.0488 (7)	0.0478 (7)	−0.0004 (6)	0.0059 (5)	−0.0002 (6)
C4	0.0349 (7)	0.0479 (7)	0.0467 (7)	−0.0058 (6)	0.0063 (5)	−0.0035 (6)
C5	0.0381 (7)	0.0444 (7)	0.0492 (7)	0.0012 (6)	0.0058 (6)	−0.0064 (6)
C6	0.0348 (7)	0.0474 (7)	0.0412 (7)	0.0029 (6)	0.0047 (5)	−0.0040 (6)
C7	0.0457 (8)	0.0416 (7)	0.0423 (7)	0.0084 (6)	0.0037 (5)	0.0053 (6)
C8	0.0618 (10)	0.0441 (8)	0.0586 (9)	0.0047 (7)	0.0012 (7)	−0.0016 (7)
C9	0.0850 (13)	0.0465 (8)	0.0688 (10)	0.0142 (8)	0.0094 (9)	−0.0072 (8)
C10	0.0837 (14)	0.0572 (10)	0.0840 (12)	0.0267 (10)	0.0286 (10)	−0.0049 (9)
C11	0.0533 (9)	0.0593 (10)	0.0910 (13)	0.0170 (8)	0.0196 (9)	−0.0030 (9)
C12	0.0434 (8)	0.0434 (7)	0.0528 (7)	0.0109 (6)	0.0080 (6)	0.0041 (6)
C13	0.0394 (7)	0.0523 (8)	0.0600 (8)	−0.0118 (6)	0.0063 (6)	−0.0060 (7)
C14	0.0947 (15)	0.0814 (13)	0.0695 (11)	−0.0406 (12)	0.0063 (10)	0.0114 (10)
C15	0.0348 (8)	0.0998 (15)	0.1037 (15)	−0.0157 (9)	0.0139 (9)	−0.0395 (13)
C16	0.0410 (7)	0.0370 (7)	0.0602 (8)	−0.0040 (6)	−0.0017 (6)	−0.0023 (6)
C17	0.0585 (9)	0.0461 (8)	0.0609 (9)	−0.0024 (7)	−0.0027 (7)	0.0011 (7)
C18	0.135 (2)	0.0608 (11)	0.0662 (12)	−0.0351 (12)	0.0065 (12)	0.0075 (9)
C19	0.0854 (15)	0.0958 (16)	0.0957 (15)	0.0206 (13)	−0.0294 (12)	0.0083 (14)
C20	0.0955 (15)	0.0829 (13)	0.0591 (10)	−0.0009 (12)	0.0053 (10)	−0.0073 (10)
C21	0.0364 (7)	0.0554 (9)	0.0570 (8)	0.0070 (6)	−0.0011 (6)	−0.0177 (7)
C22	0.0397 (7)	0.0423 (7)	0.0511 (7)	0.0031 (6)	0.0032 (6)	−0.0042 (6)
C23	0.0589 (9)	0.0560 (9)	0.0491 (8)	−0.0063 (7)	0.0001 (6)	−0.0058 (7)
C24	0.0842 (13)	0.0750 (12)	0.0471 (8)	0.0031 (10)	0.0031 (8)	−0.0055 (8)
C25	0.0908 (14)	0.0731 (12)	0.0619 (10)	0.0196 (11)	0.0284 (9)	0.0063 (10)
C26	0.0639 (11)	0.0576 (10)	0.0926 (14)	0.0064 (8)	0.0303 (10)	0.0143 (10)
C27	0.0420 (8)	0.0504 (8)	0.0717 (10)	−0.0007 (7)	0.0100 (7)	−0.0044 (8)
C28	0.0408 (7)	0.0453 (7)	0.0440 (7)	0.0028 (6)	0.0037 (5)	−0.0075 (6)
C29	0.0575 (10)	0.0680 (10)	0.0654 (10)	−0.0119 (8)	0.0015 (8)	0.0047 (9)
C30	0.0976 (15)	0.0610 (11)	0.0804 (13)	−0.0151 (10)	−0.0113 (11)	0.0129 (10)
C31	0.0965 (15)	0.0628 (11)	0.0628 (11)	0.0076 (10)	−0.0159 (10)	0.0063 (9)
C32	0.0579 (10)	0.0692 (10)	0.0612 (9)	0.0192 (8)	−0.0101 (7)	−0.0075 (8)
C33	0.0438 (8)	0.0550 (9)	0.0590 (8)	0.0043 (7)	−0.0032 (6)	−0.0037 (7)

### Geometric parameters (Å, º)

**Table:**

O—C1	1.3577 (15)	C18—H18A	0.9600
O—H0A	0.8501	C18—H18B	0.9600
N1—N2	1.3300 (16)	C18—H18C	0.9600
N1—C7	1.3544 (18)	C19—H19A	0.9600
N2—N3	1.3317 (15)	C19—H19B	0.9600
N2—C2	1.4352 (17)	C19—H19C	0.9600
N3—C12	1.356 (2)	C20—H20A	0.9600
N4—C21	1.4603 (18)	C20—H20B	0.9600
N4—C22	1.4640 (19)	C20—H20C	0.9600
N4—C28	1.4658 (17)	C21—H21A	0.9700
C1—C2	1.3940 (18)	C21—H21B	0.9700
C1—C6	1.4027 (18)	C22—C23	1.525 (2)
C2—C3	1.3865 (18)	C22—C27	1.530 (2)
C3—C4	1.381 (2)	C22—H22A	0.9800
C3—H3B	0.9300	C23—C24	1.525 (2)
C4—C5	1.4080 (19)	C23—H23A	0.9700
C4—C13	1.5351 (19)	C23—H23B	0.9700
C5—C6	1.3760 (19)	C24—C25	1.507 (3)
C5—H5A	0.9300	C24—H24A	0.9700
C6—C21	1.5204 (19)	C24—H24B	0.9700
C7—C12	1.402 (2)	C25—C26	1.515 (3)
C7—C8	1.406 (2)	C25—H25A	0.9700
C8—C9	1.361 (2)	C25—H25B	0.9700
C8—H8A	0.9300	C26—C27	1.512 (3)
C9—C10	1.404 (3)	C26—H26A	0.9700
C9—H9A	0.9300	C26—H26B	0.9700
C10—C11	1.358 (3)	C27—H27A	0.9700
C10—H10A	0.9300	C27—H27B	0.9700
C11—C12	1.412 (2)	C28—C29	1.529 (2)
C11—H11A	0.9300	C28—C33	1.540 (2)
C13—C15	1.540 (2)	C28—H28A	0.9800
C13—C14	1.542 (3)	C29—C30	1.516 (3)
C13—C16	1.551 (2)	C29—H29A	0.9700
C14—H14A	0.9600	C29—H29B	0.9700
C14—H14B	0.9600	C30—C31	1.516 (3)
C14—H14C	0.9600	C30—H30A	0.9700
C15—H15A	0.9600	C30—H30B	0.9700
C15—H15B	0.9600	C31—C32	1.512 (3)
C15—H15C	0.9600	C31—H31A	0.9700
C16—C17	1.535 (2)	C31—H31B	0.9700
C16—H16A	0.9700	C32—C33	1.521 (2)
C16—H16B	0.9700	C32—H32A	0.9700
C17—C18	1.532 (3)	C32—H32C	0.9700
C17—C20	1.534 (3)	C33—H33B	0.9700
C17—C19	1.536 (3)	C33—H33A	0.9700
			
C1—O—H0A	109.5	H19B—C19—H19C	109.5
N2—N1—C7	103.83 (11)	C17—C20—H20A	109.5
N1—N2—N3	116.48 (11)	C17—C20—H20B	109.5
N1—N2—C2	121.96 (10)	H20A—C20—H20B	109.5
N3—N2—C2	121.32 (11)	C17—C20—H20C	109.5
N2—N3—C12	102.64 (12)	H20A—C20—H20C	109.5
C21—N4—C22	112.48 (12)	H20B—C20—H20C	109.5
C21—N4—C28	116.14 (11)	N4—C21—C6	112.72 (11)
C22—N4—C28	117.98 (10)	N4—C21—H21A	109.0
O—C1—C2	124.46 (11)	C6—C21—H21A	109.0
O—C1—C6	117.36 (11)	N4—C21—H21B	109.0
C2—C1—C6	118.15 (11)	C6—C21—H21B	109.0
C3—C2—C1	121.56 (12)	H21A—C21—H21B	107.8
C3—C2—N2	118.44 (11)	N4—C22—C23	111.13 (12)
C1—C2—N2	119.93 (11)	N4—C22—C27	116.75 (12)
C4—C3—C2	121.07 (12)	C23—C22—C27	109.78 (12)
C4—C3—H3B	119.5	N4—C22—H22A	106.2
C2—C3—H3B	119.5	C23—C22—H22A	106.2
C3—C4—C5	116.94 (12)	C27—C22—H22A	106.2
C3—C4—C13	123.22 (12)	C22—C23—C24	111.85 (14)
C5—C4—C13	119.69 (13)	C22—C23—H23A	109.2
C6—C5—C4	122.89 (13)	C24—C23—H23A	109.2
C6—C5—H5A	118.6	C22—C23—H23B	109.2
C4—C5—H5A	118.6	C24—C23—H23B	109.2
C5—C6—C1	119.38 (12)	H23A—C23—H23B	107.9
C5—C6—C21	121.85 (12)	C25—C24—C23	111.37 (15)
C1—C6—C21	118.69 (12)	C25—C24—H24A	109.4
N1—C7—C12	107.66 (12)	C23—C24—H24A	109.4
N1—C7—C8	130.77 (14)	C25—C24—H24B	109.4
C12—C7—C8	121.50 (14)	C23—C24—H24B	109.4
C9—C8—C7	116.66 (17)	H24A—C24—H24B	108.0
C9—C8—H8A	121.7	C24—C25—C26	111.16 (14)
C7—C8—H8A	121.7	C24—C25—H25A	109.4
C8—C9—C10	122.13 (18)	C26—C25—H25A	109.4
C8—C9—H9A	118.9	C24—C25—H25B	109.4
C10—C9—H9A	118.9	C26—C25—H25B	109.4
C11—C10—C9	122.23 (16)	H25A—C25—H25B	108.0
C11—C10—H10A	118.9	C27—C26—C25	111.46 (15)
C9—C10—H10A	118.9	C27—C26—H26A	109.3
C10—C11—C12	117.02 (18)	C25—C26—H26A	109.3
C10—C11—H11A	121.5	C27—C26—H26B	109.3
C12—C11—H11A	121.5	C25—C26—H26B	109.3
N3—C12—C7	109.39 (12)	H26A—C26—H26B	108.0
N3—C12—C11	130.06 (15)	C26—C27—C22	112.17 (14)
C7—C12—C11	120.44 (15)	C26—C27—H27A	109.2
C4—C13—C15	111.46 (13)	C22—C27—H27A	109.2
C4—C13—C14	106.38 (13)	C26—C27—H27B	109.2
C15—C13—C14	107.68 (15)	C22—C27—H27B	109.2
C4—C13—C16	112.98 (11)	H27A—C27—H27B	107.9
C15—C13—C16	111.75 (13)	N4—C28—C29	110.65 (12)
C14—C13—C16	106.16 (14)	N4—C28—C33	116.33 (12)
C13—C14—H14A	109.5	C29—C28—C33	109.55 (13)
C13—C14—H14B	109.5	N4—C28—H28A	106.6
H14A—C14—H14B	109.5	C29—C28—H28A	106.6
C13—C14—H14C	109.5	C33—C28—H28A	106.6
H14A—C14—H14C	109.5	C30—C29—C28	112.24 (16)
H14B—C14—H14C	109.5	C30—C29—H29A	109.2
C13—C15—H15A	109.5	C28—C29—H29A	109.2
C13—C15—H15B	109.5	C30—C29—H29B	109.2
H15A—C15—H15B	109.5	C28—C29—H29B	109.2
C13—C15—H15C	109.5	H29A—C29—H29B	107.9
H15A—C15—H15C	109.5	C31—C30—C29	111.81 (16)
H15B—C15—H15C	109.5	C31—C30—H30A	109.3
C17—C16—C13	124.26 (13)	C29—C30—H30A	109.3
C17—C16—H16A	106.3	C31—C30—H30B	109.3
C13—C16—H16A	106.3	C29—C30—H30B	109.3
C17—C16—H16B	106.3	H30A—C30—H30B	107.9
C13—C16—H16B	106.3	C32—C31—C30	111.67 (16)
H16A—C16—H16B	106.4	C32—C31—H31A	109.3
C18—C17—C20	108.75 (17)	C30—C31—H31A	109.3
C18—C17—C16	112.69 (14)	C32—C31—H31B	109.3
C20—C17—C16	106.80 (14)	C30—C31—H31B	109.3
C18—C17—C19	109.63 (19)	H31A—C31—H31B	107.9
C20—C17—C19	106.13 (17)	C31—C32—C33	111.23 (16)
C16—C17—C19	112.52 (16)	C31—C32—H32A	109.4
C17—C18—H18A	109.5	C33—C32—H32A	109.4
C17—C18—H18B	109.5	C31—C32—H32C	109.4
H18A—C18—H18B	109.5	C33—C32—H32C	109.4
C17—C18—H18C	109.5	H32A—C32—H32C	108.0
H18A—C18—H18C	109.5	C32—C33—C28	111.50 (14)
H18B—C18—H18C	109.5	C32—C33—H33B	109.3
C17—C19—H19A	109.5	C28—C33—H33B	109.3
C17—C19—H19B	109.5	C32—C33—H33A	109.3
H19A—C19—H19B	109.5	C28—C33—H33A	109.3
C17—C19—H19C	109.5	H33B—C33—H33A	108.0
H19A—C19—H19C	109.5		
			
C7—N1—N2—N3	0.11 (15)	C5—C4—C13—C15	178.87 (15)
C7—N1—N2—C2	174.59 (11)	C3—C4—C13—C14	111.33 (17)
N1—N2—N3—C12	−0.10 (15)	C5—C4—C13—C14	−64.02 (18)
C2—N2—N3—C12	−174.62 (12)	C3—C4—C13—C16	−132.59 (14)
O—C1—C2—C3	178.53 (12)	C5—C4—C13—C16	52.06 (18)
C6—C1—C2—C3	0.68 (19)	C4—C13—C16—C17	71.79 (18)
O—C1—C2—N2	1.67 (19)	C15—C13—C16—C17	−54.9 (2)
C6—C1—C2—N2	−176.18 (12)	C14—C13—C16—C17	−171.99 (15)
N1—N2—C2—C3	171.18 (11)	C13—C16—C17—C18	−47.7 (2)
N3—N2—C2—C3	−14.61 (18)	C13—C16—C17—C20	−167.09 (15)
N1—N2—C2—C1	−11.86 (18)	C13—C16—C17—C19	76.8 (2)
N3—N2—C2—C1	162.35 (12)	C22—N4—C21—C6	−83.04 (16)
C1—C2—C3—C4	−0.9 (2)	C28—N4—C21—C6	136.72 (13)
N2—C2—C3—C4	175.96 (12)	C5—C6—C21—N4	−18.4 (2)
C2—C3—C4—C5	0.4 (2)	C1—C6—C21—N4	164.79 (12)
C2—C3—C4—C13	−175.04 (13)	C21—N4—C22—C23	147.68 (12)
C3—C4—C5—C6	0.3 (2)	C28—N4—C22—C23	−72.88 (15)
C13—C4—C5—C6	175.97 (13)	C21—N4—C22—C27	−85.37 (15)
C4—C5—C6—C1	−0.6 (2)	C28—N4—C22—C27	54.08 (17)
C4—C5—C6—C21	−177.38 (14)	N4—C22—C23—C24	−174.72 (13)
O—C1—C6—C5	−177.94 (12)	C27—C22—C23—C24	54.60 (18)
C2—C1—C6—C5	0.06 (19)	C22—C23—C24—C25	−55.9 (2)
O—C1—C6—C21	−1.03 (19)	C23—C24—C25—C26	55.4 (2)
C2—C1—C6—C21	176.97 (13)	C24—C25—C26—C27	−55.3 (2)
N2—N1—C7—C12	−0.07 (14)	C25—C26—C27—C22	55.49 (19)
N2—N1—C7—C8	−176.86 (14)	N4—C22—C27—C26	177.72 (13)
N1—C7—C8—C9	175.11 (15)	C23—C22—C27—C26	−54.66 (18)
C12—C7—C8—C9	−1.3 (2)	C21—N4—C28—C29	−77.80 (15)
C7—C8—C9—C10	1.3 (3)	C22—N4—C28—C29	144.21 (13)
C8—C9—C10—C11	−0.6 (3)	C21—N4—C28—C33	48.05 (17)
C9—C10—C11—C12	−0.2 (3)	C22—N4—C28—C33	−89.95 (15)
N2—N3—C12—C7	0.05 (14)	N4—C28—C29—C30	−175.64 (13)
N2—N3—C12—C11	176.20 (17)	C33—C28—C29—C30	54.80 (18)
N1—C7—C12—N3	0.01 (16)	C28—C29—C30—C31	−54.6 (2)
C8—C7—C12—N3	177.16 (13)	C29—C30—C31—C32	54.1 (2)
N1—C7—C12—C11	−176.58 (15)	C30—C31—C32—C33	−55.1 (2)
C8—C7—C12—C11	0.6 (2)	C31—C32—C33—C28	56.48 (19)
C10—C11—C12—N3	−175.62 (16)	N4—C28—C33—C32	177.89 (13)
C10—C11—C12—C7	0.2 (3)	C29—C28—C33—C32	−55.72 (18)
C3—C4—C13—C15	−5.8 (2)		

### Hydrogen-bond geometry (Å, º)

**Table:**

*D*—H···*A*	*D*—H	H···*A*	*D*···*A*	*D*—H···*A*
O—H0*A*···N1	0.85	1.88	2.618 (2)	145
